# Synthesis of superhydrophobic coatings based on silica nanostructure modified with organosilane compounds by sol–gel method for glass surfaces

**DOI:** 10.1038/s41598-023-27811-0

**Published:** 2023-01-11

**Authors:** Mohammad Ghodrati, Mehdi Mousavi-Kamazani, Zohreh Bahrami

**Affiliations:** Department of Nanotechnology, Faculty of New Sciences and Technologies, Semnan University, Semnan, Iran

**Keywords:** Chemistry, Materials science, Nanoscience and technology

## Abstract

In the present study, the superhydrophobic coating was synthesized by spherical silica nanostructures modified with organosilane compounds for glass surfaces. To optimize the conditions in terms of cost-effectiveness and create a super-hydrophobic coating with a high contact angle, the response surface method of the central composite design (CCD) model was performed for the StÖber method, and the contact angle was defined as the response surface for the model. Tetraethoxysilane (TEOS) was used as a precursor and poly(dimethylsiloxane) (PDMS) was used to modify the surface of a spherical silica nanostructure synthesized by a one-step sol–gel method using a base catalyst. The accuracy of the research was checked by the contact angle measurement test and an angle of 162° was obtained. XRD, FT-IR, EDS, SEM, DLS, and AFM analyzes were performed to investigate the synthesis of silica nanostructure. Chemical resistance was performed in acidic, neutral, and alkaline environments and the contact angles were 127°, 134°, and 90°, respectively, which indicates that the coating created on the surface glass has good chemical resistance in acidic and neutral environments.

## Introduction

Smart coatings are nanomaterials that automatically respond to changes in the environment such as heat, light, humidity, temperature, pressure, and pH. The purpose of designing such coatings for higher performance is to increase product life, and significantly reduce maintenance costs^1–7^. Due to the unique properties of nanoscale materials and the growing demand for nanomaterials in sectors such as the medical and automotive industries, research and development on nano-based coatings replace conventional polymer coatings^8,9^. Smart coatings are classified based on application, performance, reactivity, level of complexity, and manufacturing methods. Active sensing coatings include corrosion and pressure-sensitive coatings. Flame retardant coatings are penetrating and non-penetrating coatings. Anti-powder and antibacterial coatings are known as activating coatings. Easy-to-clean coatings include self-cleaning and anti-graphite coatings. Smart window coverings are optically active coatings. Other coatings are anti-fingerprint, anti-reflective, anti-freeze, and anti-fog^10^. Ultra-waterproof coatings are an important category of smart coatings that have received a lot of attention due to their properties. These coatings can be used in any of the above coatings due to their unique properties. For example, due to biodegradation, they can be used in self-healing and antibacterial coatings^11–14^, due to morphology and size in self-cleaning and anti-corrosion coatings^15–19^, and due to their chemical properties in antifreeze and anti-vapor^19–21^.

Superhydrophobic surfaces are known for two important properties, the first is the surface roughness at the micro and nanoscales and the second is the complex structure. Therefore, synthesis methods such as electrochemical deposition^22^, CVD^23^, layer-by-layer (LBL) deposition^24^, hydrothermal^25^, and sol–gel can^26^ be used to develop and fabricate the mentioned properties. The sol–gel method consists of two stages of hydrolysis and condensation. The raw materials used are silane and metal alkoxides. Among the advantages of the sol–gel method are low-temperature synthesis, high purity, precise control of particle size and distribution, and the possibility of making new crystalline and non-crystalline materials^27,28^. Rough surfaces can be created with the help of SiO_2_^29^, Al_2_O_3_^30^, and CuSO_4_^31^, and with the help of hydrophobic agents such as poly(dimethylsiloxane) (PDMS)^32^, hexadecyltrimethoxysilane (HDTMS)^33^, surfaces with low surface energy can be made.

The purpose of using the response surface (method) is to design an experiment that examines the possibility of a quadratic interaction between the parameters in the experiment. With the help of the method CCD, it is possible to improve, optimize the process, and also to diagnose the problems and weak points of the process, as a result, to design a process resistant to external influences that produce a suitable product^34^.

In this research, for the first time, the Design-Expert software and the response surface method of the central composite design model (CCD) were used to synthesize a superhydrophobic coating on the glass surface to optimize the parameters of the Stöber process. The contact angle of the water drop was used as the response surface. The selected parameters include deionized water as a hydrolysis agent, ethanol as a solvent, ammonia as a catalyst, and polydimethylsiloxane as a surface modification agent. In this method, experimental design is done by determining the actual levels and coding levels for each parameter (i.e. + 1 for high levels, zero central levels, and -1 for low levels).

## Experimental

### Materials

All chemical materials and compounds used in this study, including tetraethyl orthosilicate (TEOS) (Si(OC_2_H_5_)_4_), poly(dimethylsiloxane) (PDMS) ((C_2_H_6_OSi)_n_), ammonia (NH_3_OH), ethanol (C_2_H_5_OH), and hydrochloric acid (HCl) were purchased from Merck. All chemicals materials used were highly purified and consumed without purification.

### Instruments

The contact angle was measured by Jikan CAG-20. XRD (X-ray diffraction) pattern was studied by Philips-X PertPro device using Ni-filtered Cu Kα radiation. FT-IR (Fourier transform infrared) analysis was performed with Magna-IR, spectrometer 550 Nicolet with a resolution of 0.125 cm^−1^ in KBr pellets ranging from 400 to 4000 cm^−1^. The light transmittance (transparency) by PHYSTEC—UVS 2500 was investigated. EDS (energy dispersion spectroscopy) analysis was performed using a Philips XL30 microscope device. MIRA3 FEG-SEM was used to record FESEM (field-emission scanning electron microscope) images. Imaging of surface topography by NT-MDT, SOLVER, Nova-Tech was done. Particle size distribution and zeta potential scattering properties were performed by Malvern Zs90.

### Synthesis of SiO_2_ nanostructures

Silica nanostructures were synthesized by two methods: sol–gel and hydrothermal. Silica sol was prepared by the StÖber method, which is a subset of the sol–gel process.

#### Sol–gel

In this method, four ingredients are used to prepare the silica sol. First, 10 ml of distilled water, which acts as a hydrolyzer, was mixed with 8 ml of ethanol (solvent) for 5 min. Then, 2 mL of TEOS was added to the solution and stirred for 5 min. 9 mL of ammonia (as a base catalyst) was added dropwise to the solution until the color of the solution changed from clear to white, and then the stirring of the solution was continued for 30 min (Sample 1). All steps are performed at room temperature. The values and conditions of other samples are listed in Table 2.

#### Hydrothermal

Once the sol was made, it was transferred to an autoclave and placed in an oven at 70 °C for 4 h to perform the compaction process at constant pressure and temperature. Then, the aging time was considered for 7 days before covering.

#### Coating of silica nanoparticles and heat treatment

There are various methods for creating a super-water-repellent coating on the glass surface, such as immersion coating, rotating coating, and spraying. The rotational coating was chosen to create a uniform nanometer coating with the glass slide on the turntable and a vacuum pump used to hold the glass in place at the time. Then, using a 0.5 ml syringe, silica gel was poured on the side of the glass. The turntable rotated for 20 s at a speed of 2000 rpm. The glass slide is then placed in the oven at 100 °C for 3 h.

#### Surface modification and heat treatment

**Figure 1:**
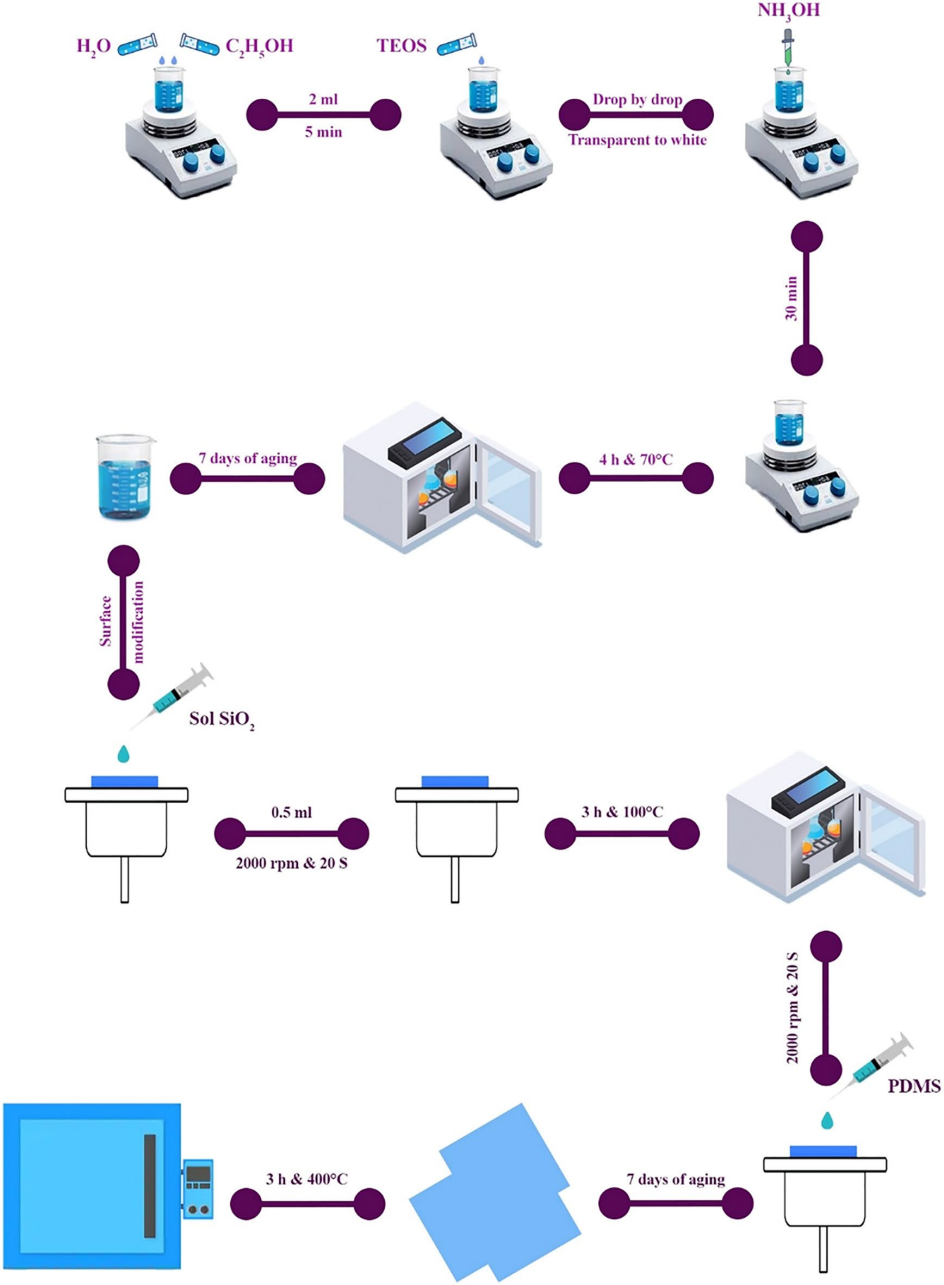
Superhydrophobic coating synthesis process.

PDMS was chosen for surface energy reduction and surface modification due to its low surface energy. The spin coating method was used to modify the surface of the super-hydrophobic coating. First, 40 $\mathrm{\mu l}$ of PDMS was poured onto glass slides and the glass slide was rotated for 20 s at a speed of 1500 rpm (Sample 1) until a uniform coating was formed. The reaction conditions for other samples are presented in Table 2. After correcting the surface, the aging time was considered for 7 days. The aging process is due to the creation of Si–O-Si bonds. The glass was then placed in a furnace at 400 °C for 3 h. The synthesis process of the superhydrophobic coating is presented in Fig. 1. Finally, the necessary tests were performed to check the properties.

## Results and discussion

### Design-expert and analysis of results

There are two factors for making a super-waterproof coating: rough surface and rough surface correction. The StÖber method is one of the methods for synthesizing silica nanoparticles to make a rough surface. The purpose of designing the experiment is to optimize the parameters of the StÖber method to create a suitable rough surface and a surface modifying agent for the superhydrophobic coating. Finally, TEOS was investigated as a fixed precursor and the effect of deionized water (A), ethanol (B), ammonia (C), and PDMS (D). The central composite design (CCD) method was used for the design.

In this method, the experimental design is performed by specifying the actual levels and coding levels for each parameter (i.e. for high levels + 1, central levels zero, and low levels − 1). Actual and coded levels are shown in Table 1. The experimental design matrix with the levels encoded by the software is shown in Table 2. The obtained contact angle was entered into the software as a response. The actual and coded performance levels were used to conduct practical tests. Surface (− 1111) (experiment no. 10) with a contact angle of 110.4° was the lowest and surface (0000) (experiment no. 6) with a contact angle of 166.5° was the highest.

**Table 1:** Actual levels and coded reaction parameters.

Factors	Code and level		
	− 1	0	+ 1
A: Distilled water	8	10	12
B: Ethanol	10	12	14
C: Ammonia	6	9	12
D: Polydimethylsiloxane	20	30	40

**Table 2:** Experimental design matrix with coded surfaces.

Run	Factors				WCA actual
	A	B	C	D	
1	0	− 2	0	0	125.7
2	− 1	1	− 1	− 1	119.2
3	− 1	1	1	− 1	132.9
4	− 1	− 1	1	1	142.4
5	2	0	0	0	130.7
6	0	0	0	0	166.5
7	0	0	0	0	165.5
8	− 1	1	− 1	1	112.7
9	− 1	− 1	− 1	− 1	117.3
10	− 1	1	1	1	110.4
11	− 2	0	0	0	118.5
12	0	0	0	0	164.5
13	0	0	0	0	165.8
14	0	2	0	0	121.5
15	1	1	1	1	125.8
16	0	0	0	− 2	137.9
17	− 1	− 1	1	− 1	136.2
18	1	1	− 1	1	139.7
19	1	1	1	− 1	146.1
20	1	− 1	− 1	1	145.1
21	0	0	0	0	166.3
22	0	0	2	0	132.8
23	1	− 1	− 1	− 1	112.4
24	0	0	0	2	142.4
25	0	0	0	0	165.5
26	1	− 1	1	− 1	121.3
27	1	− 1	1	1	132.3
28	1	1	− 1	− 1	141.4
29	0	0	− 2	0	127.1
30	− 1	− 1	− 1	1	146.8

#### ANOVA analysis

According to the obtained statistical data and the ANOVA table, it is a good model that has the following two conditions:

1. p-value < 0/05
2. In the selected model, R^2^ should be closer to one (Table 3).

**Table 3:** Quality of fitted to experimental data.

R^2^	0.9980
Adjusted R^2^	0.961
Predicted R^2^	0.9895
Adeq precision	71.9315

R^2^ checks the quality of the experimental data with the model and the best value is one.

Adj- R^2^ is the modified value of R^2^, which also takes into account the degree of freedom (number of factors).

The Predicted R^2^ of 0.9897 agrees with the adjusted R^2^ of 0.9961; That is, the difference is less than 0.2.

Adeq Precision measures the signal to noise ratio. A ratio greater than 4 is desirable. Your ratio of 71.932 indicates an adequate signal. This model can be used to navigate the design space.

Statistical data were analyzed using the response level method and regression equation:

Regression equation in encrypted units:

$$\begin{gathered} {\text{CA }} = { 165}.{683 } + { 2}.{942}0{8}*{\text{A }} + \, - {1}.{41625}*{\text{B }} + \, 0.{994583}*{\text{C }} + { 1}.{557}0{8}*{\text{D }} + { 6}.{84688}*{\text{AB }} + \, - {2}.{44}0{63}*{\text{AC }} + \, 0.{938125}*{\text{AD }} \hfill \\ + - 0.{533125}*{\text{BC }} + \, - {8}.{14938}*{\text{BD }} + \, - {4}.{97438}*{\text{CD }} + \, - { 1}0.{1651}*{\text{A}}^{{2}} + \, - {1}0.{4151}*{\text{B}}^{{2}} + \, - {8}.{83635}*{\text{C}}^{{2}} + \, - {6}.{29135 }*{\text{ D}}^{{2}} \hfill \\ \end{gathered}$$

The quadratic response level model is used to evaluate the effectiveness of the parameters and the accuracy of the model.

The model F value of 528.44 indicates that the model is acceptable. There is only a 0.01% chance that an F value of this magnitude will occur due to the disturbance.

P-values less than 0.0500 indicate that the model parameters are significant. In this case, A, B, C, D, AB, AC, AD, BD, CD, A^2^, B^2^, C^2^, and D^2^ are acceptable parameters. Values that have p values greater than 0.0500, such as the BC parameter, indicate that this parameter does not affect the test conditions. Changing the model may improve your model if many model parameters are not affected.

The Lack of Fit F-value of 3.16 indicates that a mismatch to a pure error is not acceptable. There is an 10.78% chance that a mismatch of the F-value of this magnitude will occur due to a disturbance. Non-significant disproportion is good because we want the model to fit Table 4.

**Table 4:** Analysis of variance.

Source	Sum of squares	df	Mean square	F-value	p-value	
Model	9135.54	14	652.54	528.44	< 0.0001	Significant
A-H_2_O	207.68	1	207.68	168.19	< 0.0001	Significant
B-EtOH	48.17	1	48.17	39.01	< 0.0001	Significant
C-NH_3_	24.40	1	24.40	19.76	0.0005	Significant
D-PDMS	58.28	1	58.28	47.20	< 0.0001	Significant
AB	748.02	1	748.02	605.77	< 0.0001	Significant
AC	95.06	1	95.06	76.98	< 0.0001	Significant
AD	14.06	1	14.06	11.39	0.0042	Significant
BC	4.41	1	4.41	3.57	0.0783	Not significant
BD	1062.76	1	1062.76	860.65	< 0.0001	Significant
CD	396.01	1	396.01	320.70	< 0.0001	Significant
A^2^	2842.02	1	2842.02	2301.54	< 0.0001	Significant
B^2^	2983.34	1	2983.34	2415.98	< 0.0001	Significant
C^2^	2144.23	1	2144.23	1736.45	< 0.0001	Significant
D^2^	1085.76	1	1085.76	879.28	< 0.0001	Significant
Residual	18.52	15	1.23			
Lack of fit	15.99	10	1.60	3.16	0.1078	Not significant
Pure error	2.53	5	0.5057			
Cor total	9154.07	29				

#### Diagnostics diagrams

**Figure 2:**
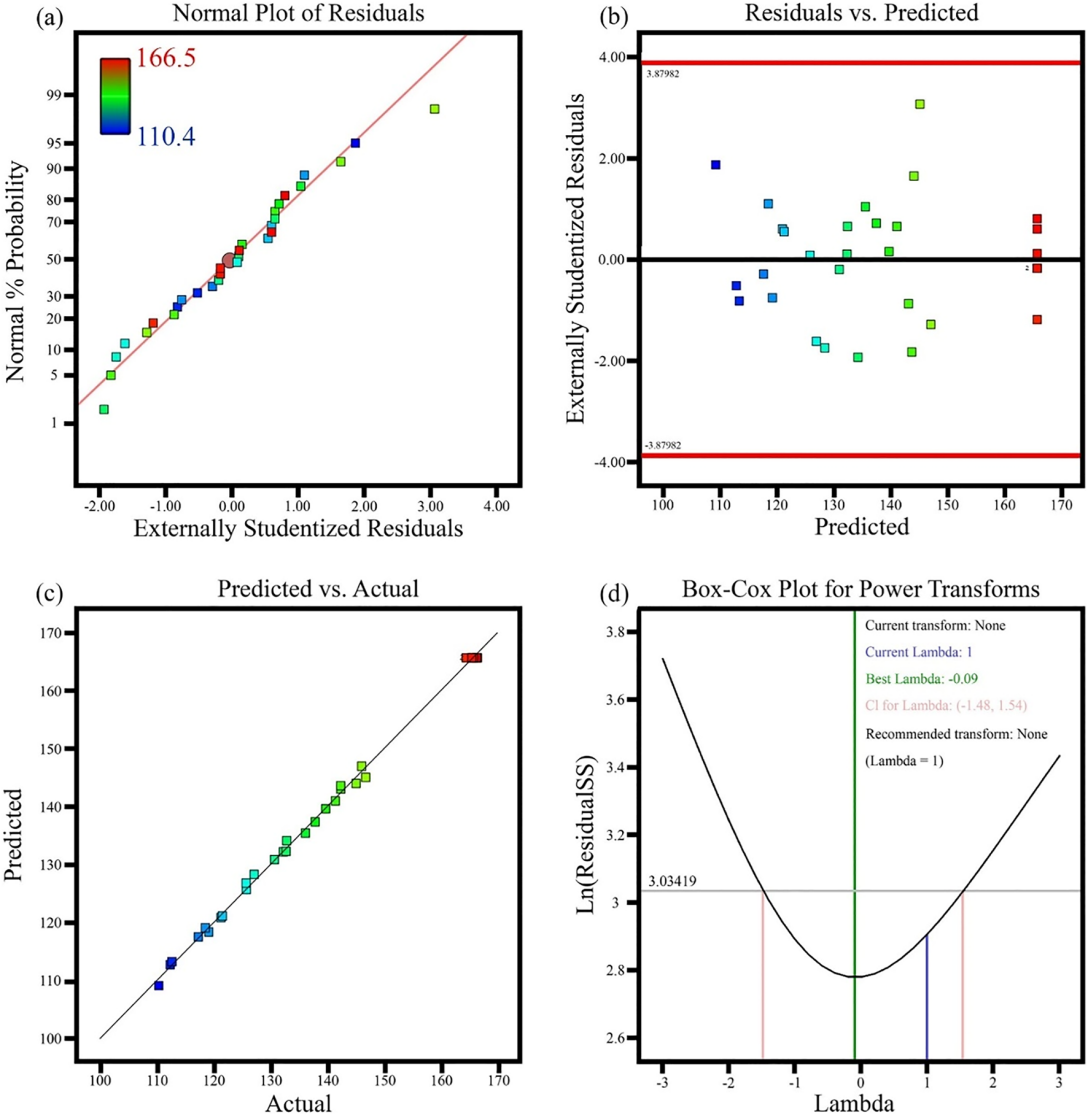
Diagnostics diagrams (**a**) normal probability, (**b**) residuals vs. predicted, (**c**) predicted vs. actual and (**d**) Box-Cox plot for power transforms.

To troubleshoot the results obtained from the software the four graphs of normal probability, residuals vs. predicted, predicted vs. actual and Box-Cox plot for power transforms are used. In Fig. 2a, the normal probability diagram shows that the residuals follow a normal distribution, therefore they follow a straight line. Even with normal data, expect some scatter. Figure 2b (residuals vs. predicted) indicates that the residuals are bullish against the predicted response values. This plot tests the assumption of constant variance. The graph should have a random scatter and according to the graph the data follows a random scatter. Figure 2c shows predicted vs actual. A graph of the predicted response values versus the actual response values. The purpose is to detect a value, or group of values, that are not easily predicted by the model. The Box-Cox chart is used to determine the strength of metamorphism consistent with experimental data (Fig. 2d). The blue line in the diagram shows the model change and the green line shows the best lambda value. The red line indicates the 95% confidence interval associated with the best amount of lambda. It is said that a model is qualified for the blue conversion line between the red lines and the green line on the conversion curve to form a black and white curve. The graph shows that the blue transition line between the green line and the red line shows that the model matches the experimental results.

#### Influence of single variables on contact angle

**Figure 3:**
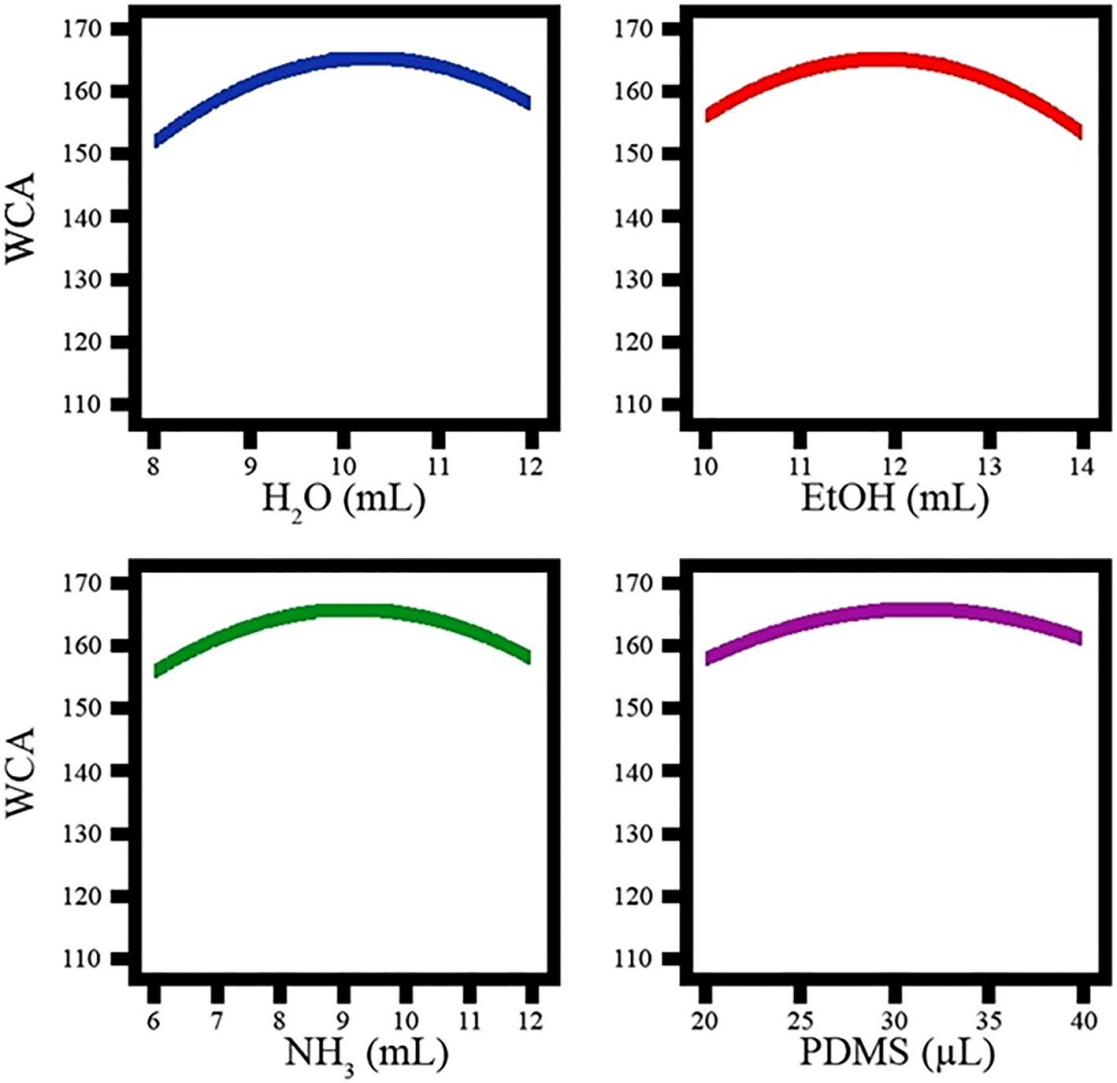
Influence of single variables on contact angle.

Figure 3 shows the effect of the selected parameters on the contact angle. The graph follows a certain pattern for all parameters. With increasing water volume from 8 to 10, the contact angle gradually increased from 152.34° to 165.83°. Subsequently, with an increase from 10 to 12, the contact angle decreased from 165.83° to 158.45°. For the parameters of ethanol, ammonium hydroxide, and PDMS, the same changes occurred, i.e. the contact angle increased from − 1 to zero and decreased from zero to + 1.

#### The effect of binary variables on the contact angle

**Figure 4:**
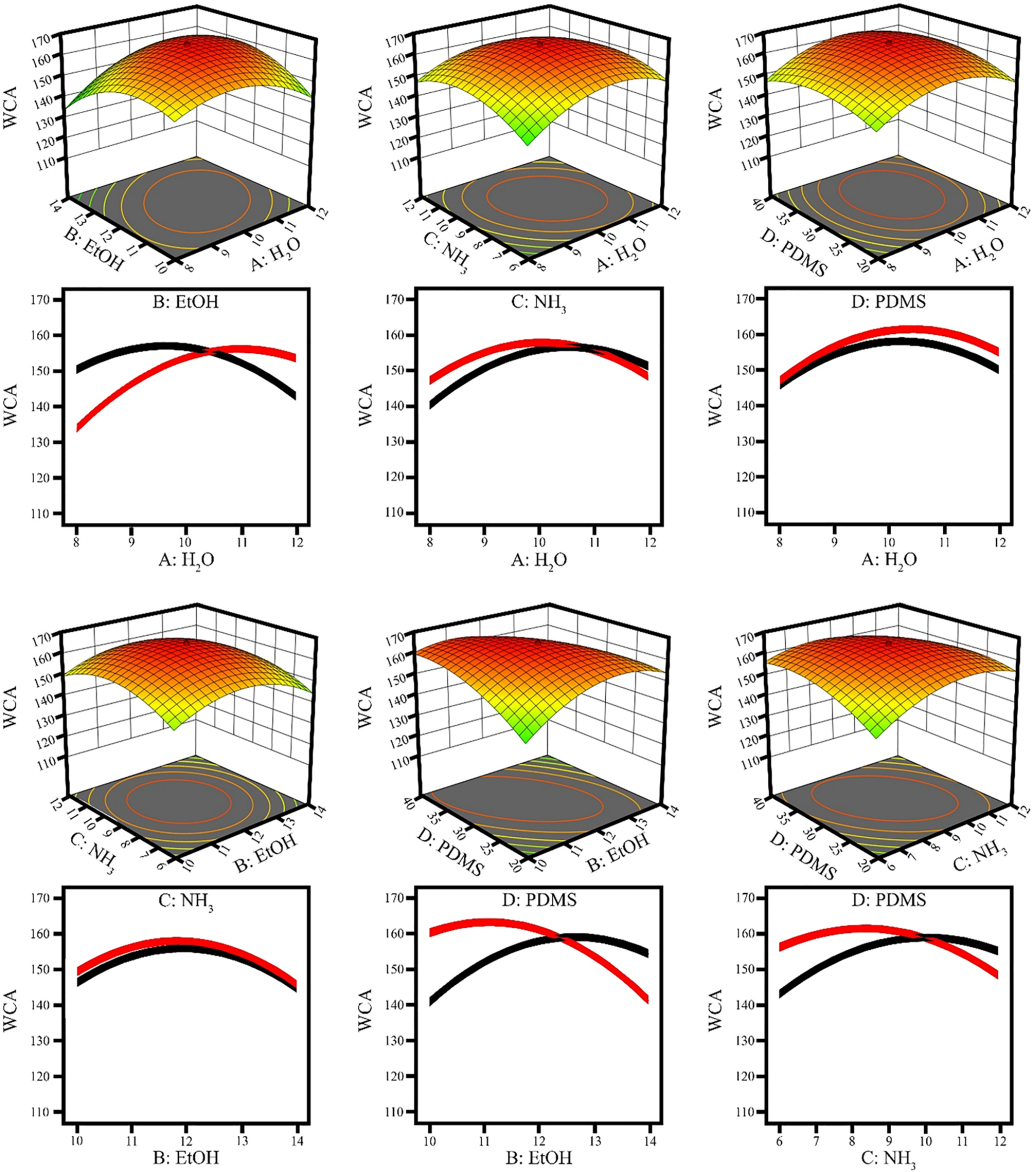
The effect of binary variables on the contact angle.

In Fig. 4, the interaction and three-dimensional diagrams of the model are obtained to estimate the interaction between the variables and the contact angle, while the other variables are kept at their zero levels and the others change in the experimental range. In Fig. 4, as can be seen, the interaction of the binary parameters with each other is like the effect of the parameters individually. The difference between the interaction of the binary parameters with each other is in the angle between the two diagrams. The higher the angle of the two graphs, the greater the interaction, and if they are parallel, they have less or no interaction. According to the ANOVA table, the BC parameter is unacceptable, and the angle between the parameter BC is very small and they are almost parallel.

#### Process design optimization

**Figure 5:**
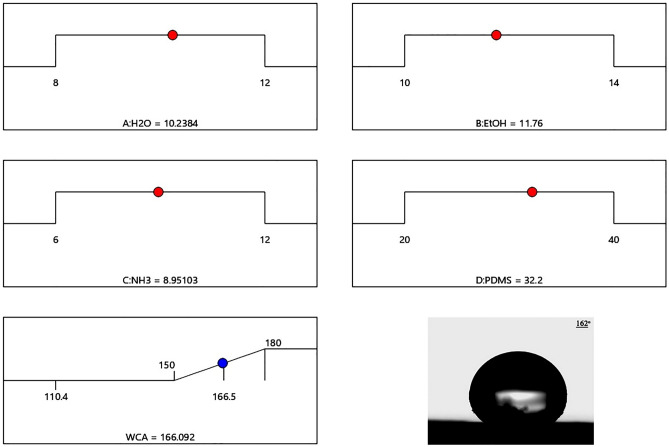
Optimal values of process parameters.

One of the purposes of test design is to optimize process parameters to obtain the highest contact angle. The size of the contact angle was determined to be 162°. According to the CCD design, the optimal conditions for the preparation of SiO_2_ sol are shown in Fig. 5. The experimental contact angle size for the optimized sample is compared with the predicted contact angle in Fig. 5. The results showed that the size of the experimental contact angle corresponds well with the predicted contact angle and shows that the CCD surface response surface method is an efficient method for preparing ultra-waterproof coatings with a contact angle above 160°.

### Identify the optimized sample

Optimized sample according to statistical data analysis, as mentioned earlier, was prepared by StÖber method with TEOS as precursor, deionized water of hydrolyzing agent, solvent ethanol, ammonium hydroxide catalyst, and PDMS as a hydrophobic agent. As a result, the effect of experimental parameters on superhydrophobic coatings was investigated.

#### Optimized sample contact angle

**Figure 6:**
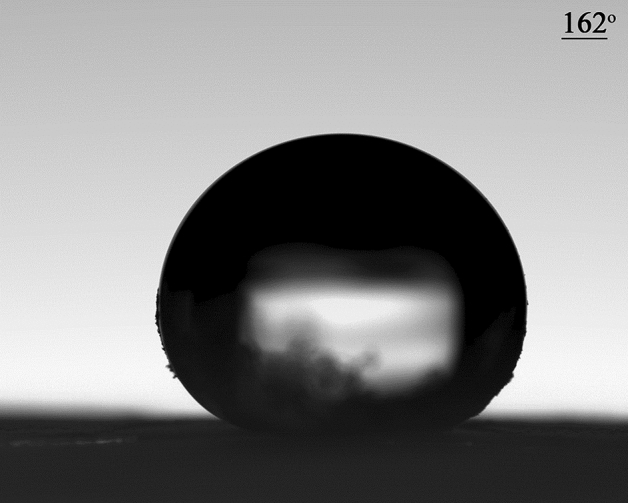
Contact angle between the drop and the glass surface.

The contact angle is the angle between the surface on which the liquid is located or the point of connection of the liquid on the surface. Static and dynamic contact angles are of its types. The method of measuring the contact angle is called the baseless droplet method. Can be used. The contact angle (CA) of the sliding angle (SA) obtained for the optimized sample is 162° and 5°, respectively (Fig. 6).

#### XRD studies

**Figure 7:**
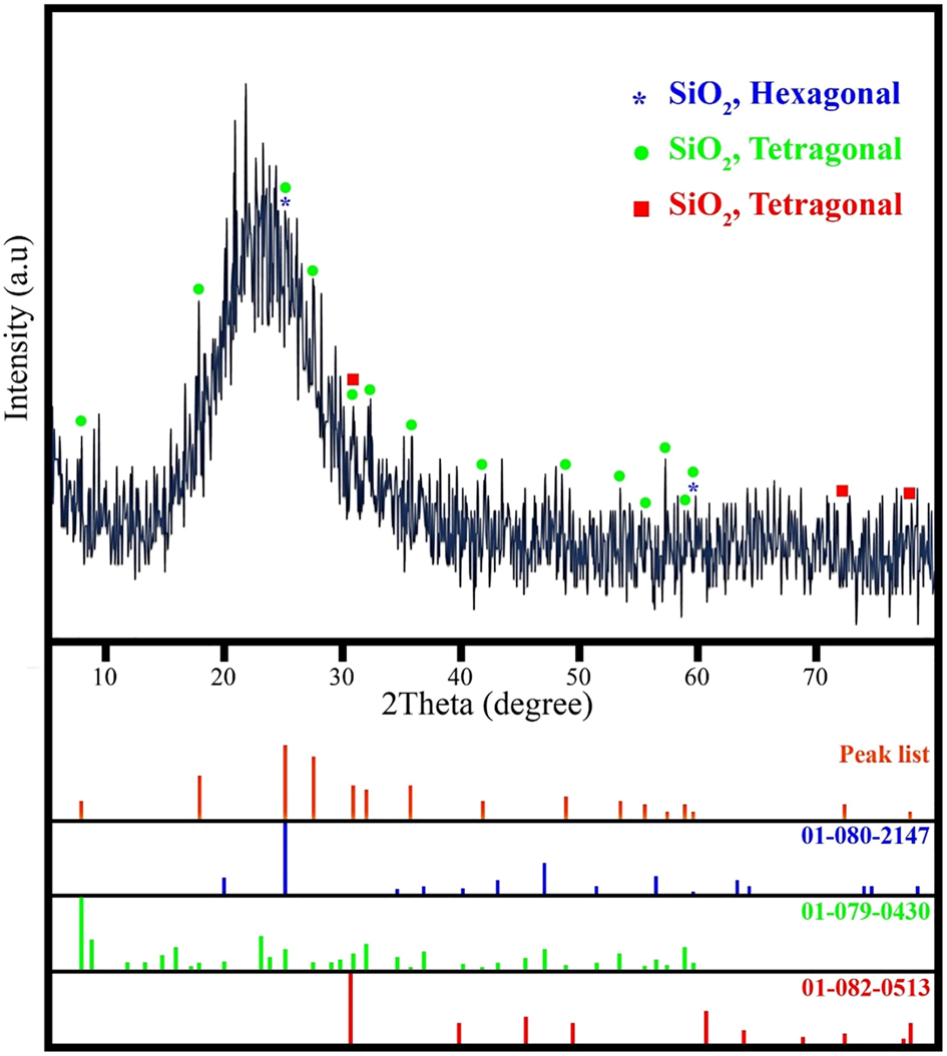
XRD pattern of the optimized sample.

Figure 7 shows the X-ray diffraction pattern of nanoparticles prepared by the StÖber method. As shown in the figure, no diffraction peaks are observed except for broadband with a 24-degree center (JCPDS No. 0085-29) which represents a completely amorphous structure. But using Highscore plus software, it is shown that a small part of the sample has a crystalline structure. The marked peaks are related to hexagonal (JCPDS number 2147-080-01) and quadrilateral (JCPDS number 0430-079-01) and (JCPDS number 0513-082-01) crystal structures.

#### FTIR spectrum

**Figure 8:**
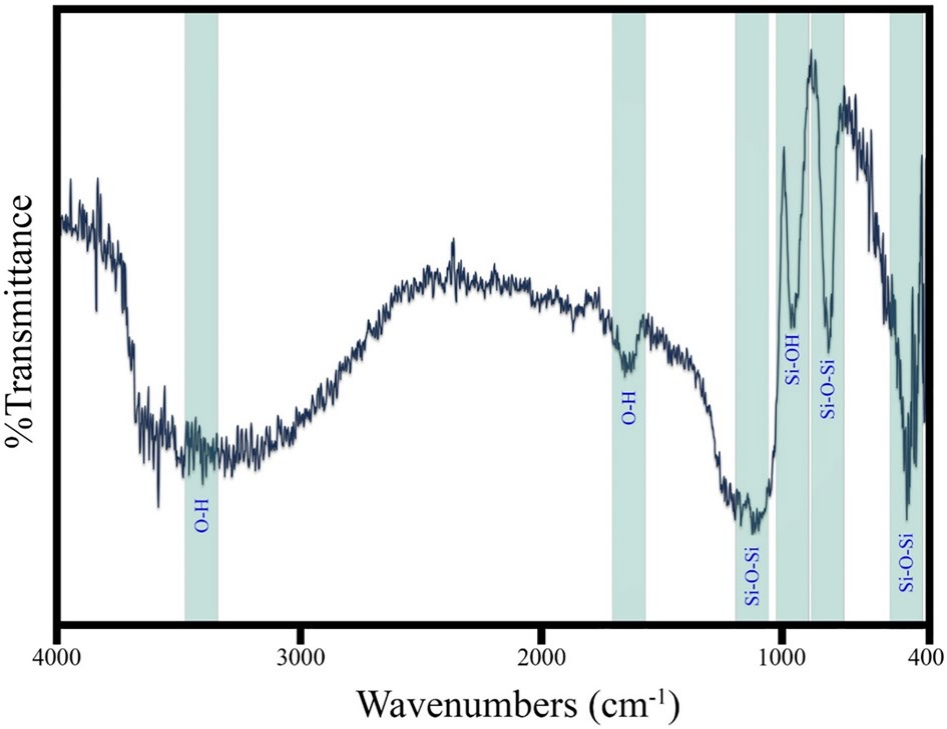
FT-IR spectrum of the optimized sample.

Infrared spectroscopy (FT-IR) was performed at room temperature to investigate the chemical bonds created in the optimized sample. As shown in Fig. 8, the peaks of 3440 cm^−1^ and 1624 cm^−1^ are symmetrical tensile vibration and flexural vibration of the O–H bond, respectively, due to the incomplete density of the silanol group^35,36^. The range of 400–1350 cm^−1^, known as the fingerprint area, indicates silicon bonds. The peak 1095 cm^−1^ and 808 cm^−1^ represent the symmetric and asymmetric tensile vibrations of the Si–O–Si bond and the peak 466 cm^−1^ represents the flexural vibrations of the Si–O–Si^37,38^. The peak of 947 cm^−1^ is due to the flexural vibrations of the Si–OH bond^39^. As can be seen, the aging period causes the formation of a Si–O–Si tensile and bending bond in the sample. This creates a resistant coating on the surface of the glass.

#### SEM images

**Figure 9:**
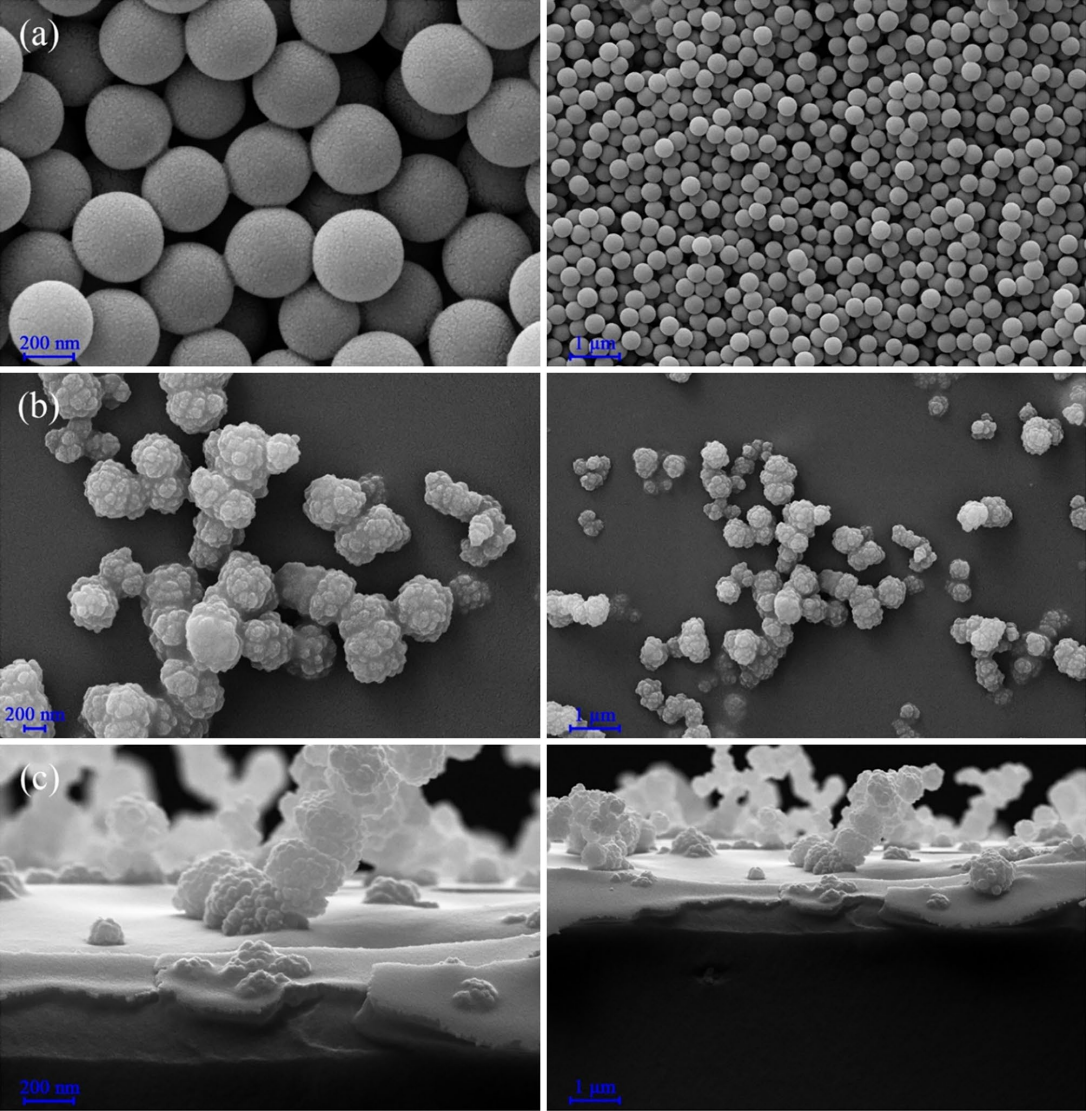
(**a**) SEM images of the optimized sample, (**b**) SEM images of the glass surface, and (**c**) cross-sectional SEM images of silica-coated glass.

Scanning electron microscope images of the optimized sample at different magnifications are shown in Fig. 9. As seen in Fig. 9a, the nanostructures have a spherical morphology with a size of about 250 nm. The reason for the growth of nanoparticles is due to the use of the hydrothermal method to create a suitable uneven surface on the glass surface. The shape and size of the nanostructure have an essential effect on creating a super hydrophobic coating. As can be seen, the spherical nanostructures have made a rough surface on the glass. Figure 9b,c show the scanning electron microscope image of the surface and cross-section. The roughness of the glass surface indicates a rough surface for the super-hydrophobic coating. The growth process of this superhydrophobic coating is island-layer, which is a state between layer-by-layer growth and island growth, one or more monolayers are formed and then the islands are completed. Another name for this growth process is Stransky-Kristanov. In this growth mode, a mismatched network may be formed between the coated layer and the substrate. The grain size of the thin layer that is formed on the substrate depends on the speed and temperature of the layer. The thickness of this superhydrophobic coating is reported to be 1.06 µm.

#### EDS studies

**Figure 10:**
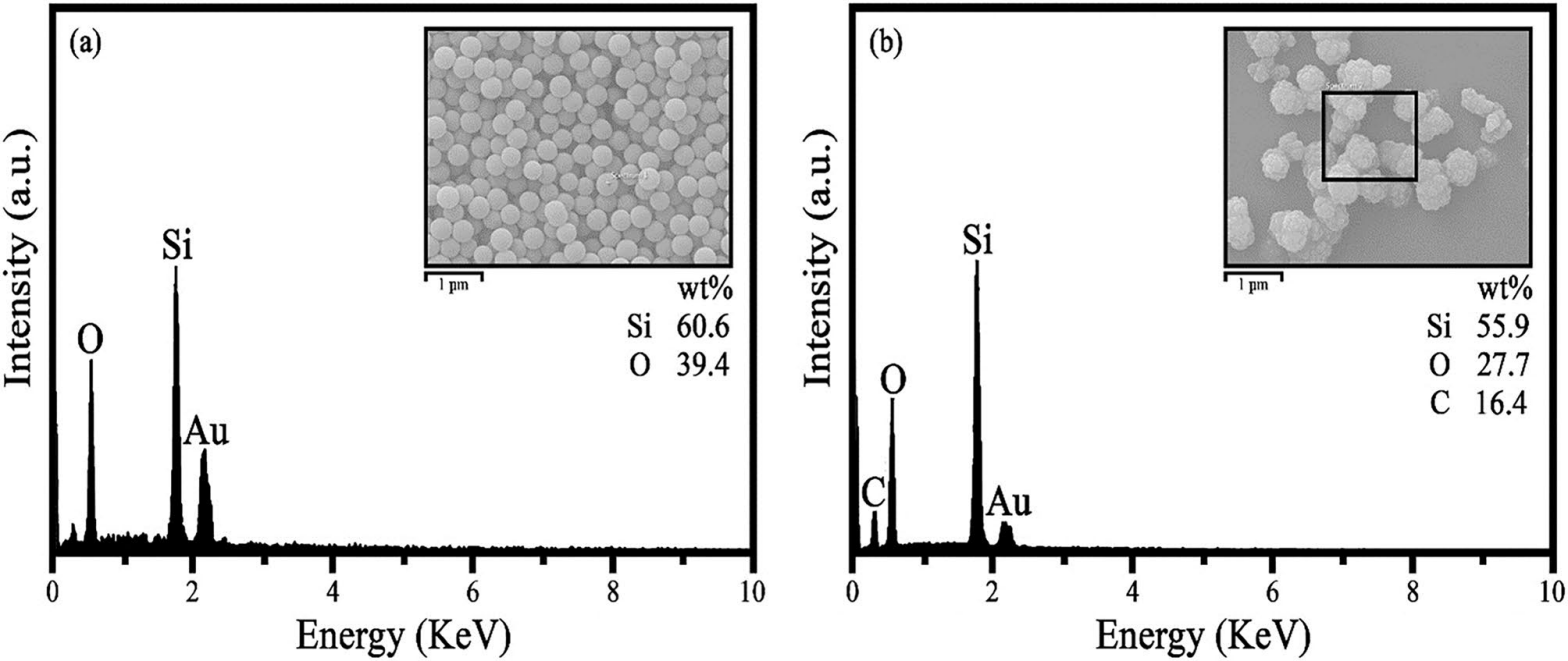
EDS for the (**a**) optimized sample and (**b**) glass surface.

Figure 10a shows the X-ray energy diffraction spectrum for the optimal sample. As shown in the figure, the purely synthesized optimized sample is composed of silicon and oxygen elements. Figure 10b shows the X-ray diffraction spectrum from the glass surface. Due to the use of PDMS as a hydrophobic agent on the glass surface, in addition to silicon and oxygen, carbon is also seen. The gold peak seen in the figure is due to the conductivity of the surface for SEM analysis.

#### AFM images

**Figure 11:**
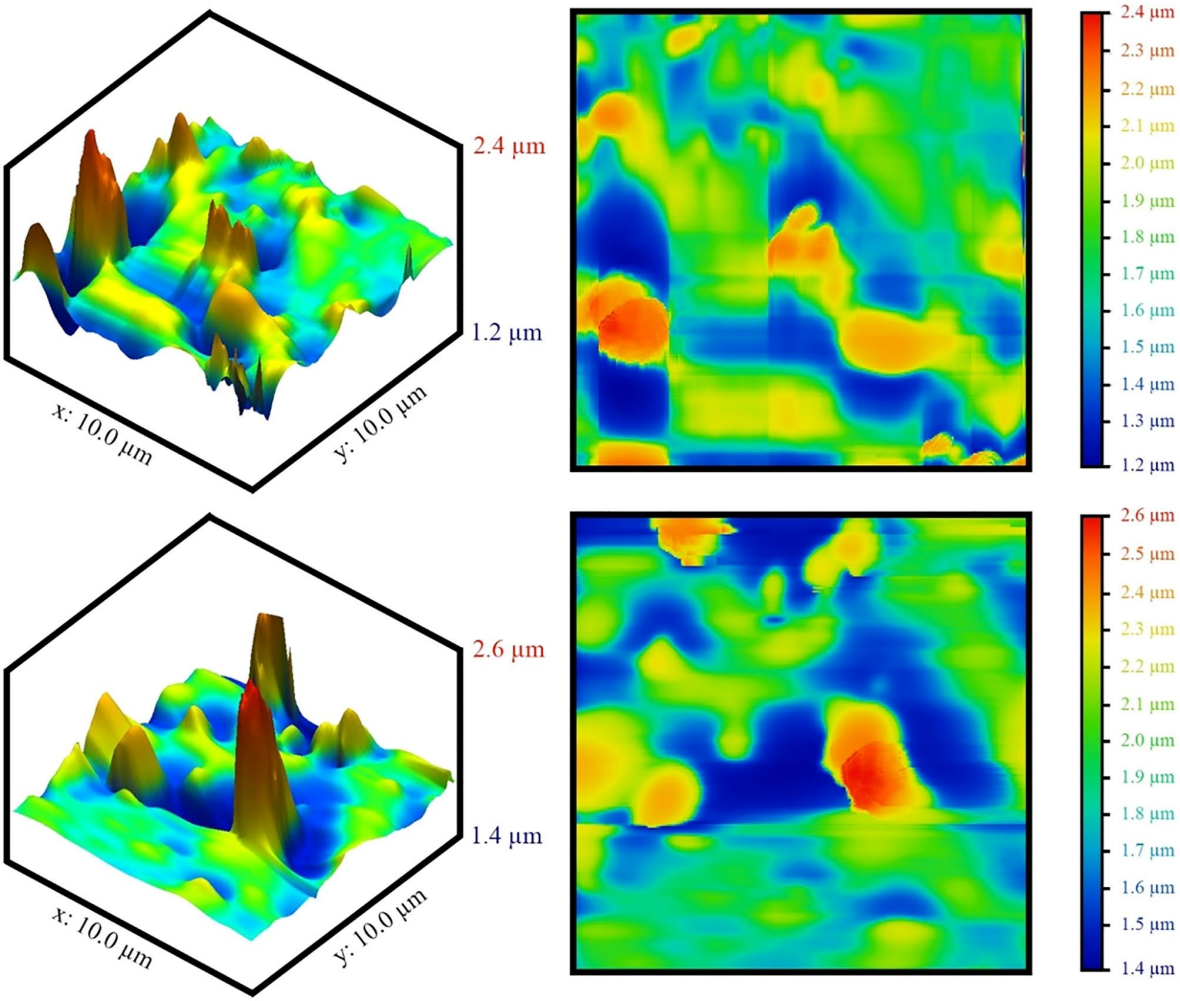
AFM images of the optimized sample.

Atomic force microscopy images of the mean square root roughness for the optimized sample are estimated in Fig. 11. As can be seen in the figure, rough surface roughness is created on the glass surface to create a super-hydrophobic coating. The maximum and minimum of these surface roughnesses were measured at 2.6 and 1.2 μm, respectively. The root means square roughness for the sample optimized by Gwyddion software was calculated to be 0.121 μm.

#### DLS analysis

**Figure 12:**
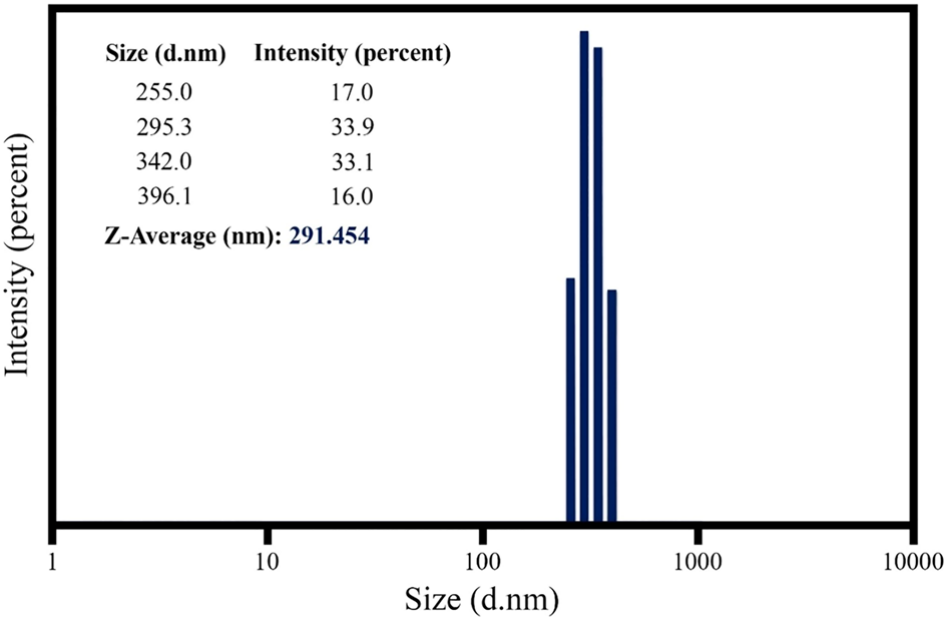
Size distribution of the optimized sample.

As can be seen in the SEM images, the optimized sample has the same particle size distribution and DLS analysis was performed to determine the particle size distribution range. The particle size range is between 255 and 396.1 nm and, as shown in Fig. 12, the particle size distribution diagram is very narrow. The average particle size is 291.456 nm. Coating thickness and surface roughness depend not only on particle size but also on size distribution. Therefore, as much as the particle size is properly distributed, the surface roughness increases and becomes uniform.

#### Chemical resistance test

**Figure 13:**
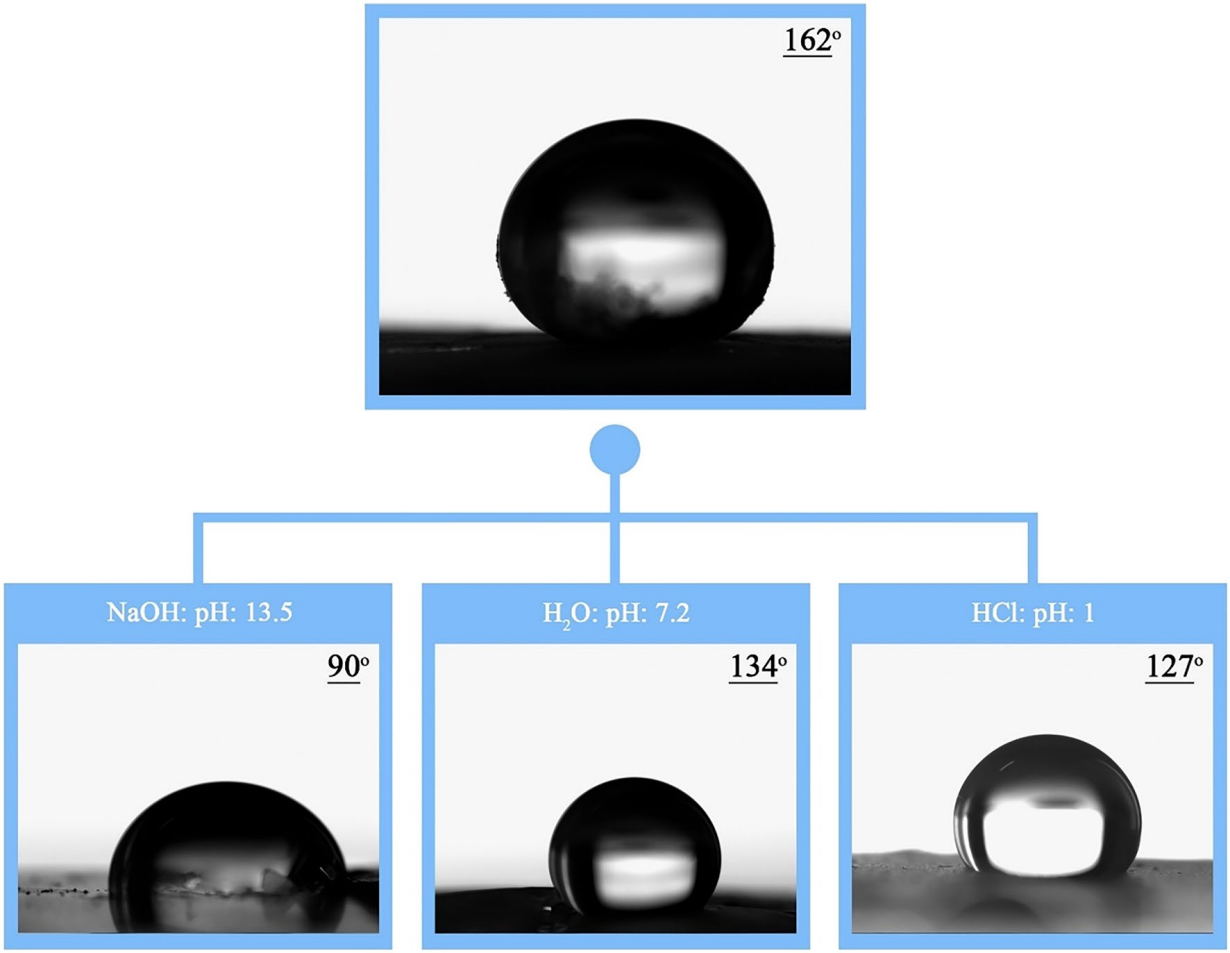
Effect of pH on the contact angle of the optimized sample.

The stability of superhydrophobic coating was investigated in three media: acidic, neutral, and alkaline. 1 M KOH and 1 M HCl solutions were prepared for the resistance of the super-hydrophobic coating in alkaline and acidic environments, respectively. Figure 13 shows the effect of different environments on the contact angle. The superhydrophobic coating was immersed in 10 ml for 24 h. It was then dried at room temperature. As can be seen in the figure, the play environment (pH: 13.5) has a great impact on the coating so that the drop contact angle of the super-hydrophobic range reaches the boundary between hydrophobic and hydrophilic, in which case the result can be the game environment causes corrosion of the coating and the use of this coating in play environments is not recommended. The contact angle obtained from the acidic environment (pH: 1) indicates that the coating has good resistance and this feature can be very effective in coating against acid rain. The use of deionized water is used to accurately assess the strength of the coating when in contact with water. Therefore, to prevent the effect of temperature on the coating, this test was performed at a temperature of 25°. The contact angle resulting from the immersion of the coating in deionized water indicates the very good resistance of the coating in this environment.

## Conclusions

The Design-Expert software was used to provide a superhydrophobic coating to optimize the test conditions to save time and money and obtain the best answer for the test parameters. The central composite design method was used to perform all possible experiments to obtain the best result. Silica nanostructures were synthesized by the sol–gel-hydrothermal method according to the number of experiments and designed values and the rotational coating was used to create a thin layer on the glass. The contact angle of each experiment was examined as the answer by the software and the optimized values of the experimental parameters were used to synthesize the optimized sample. The optimized sample was identified by the relevant analyzes and the following results were obtained: To prove the superhydrophobic coating, the contact angle between the drop and water was examined and an angle of 162° was obtained. The powder obtained from silica sol was examined and according to XRD analysis, it is a large part of the amorphous nanostructure. And according to the FT-IR spectrum, the Si–O–Si bond was detected. Silica nanostructures have a uniform spherical morphology with a particle size between 255 and 396 nm and the thickness of this nanostructure is 1.06 μm. The surface roughness on the glass surface indicates a super-hydrophobic coating. According to SEM images and DLS analysis, the optimized sample has a uniform size distribution and according to the amount of zeta potential obtained, it has a desirable dispersion property. For chemical resistance, the coating was placed in three environments acidic, neutral, and alkaline for 24 h, and according to the contact angle obtained, it can be concluded that the coating supplied superhydrophobic in neutral and acidic environments more than in other environments.

## Data Availability

The datasets used and analyzed during the current study available from the corresponding author on reasonable request.
